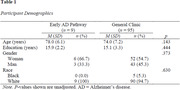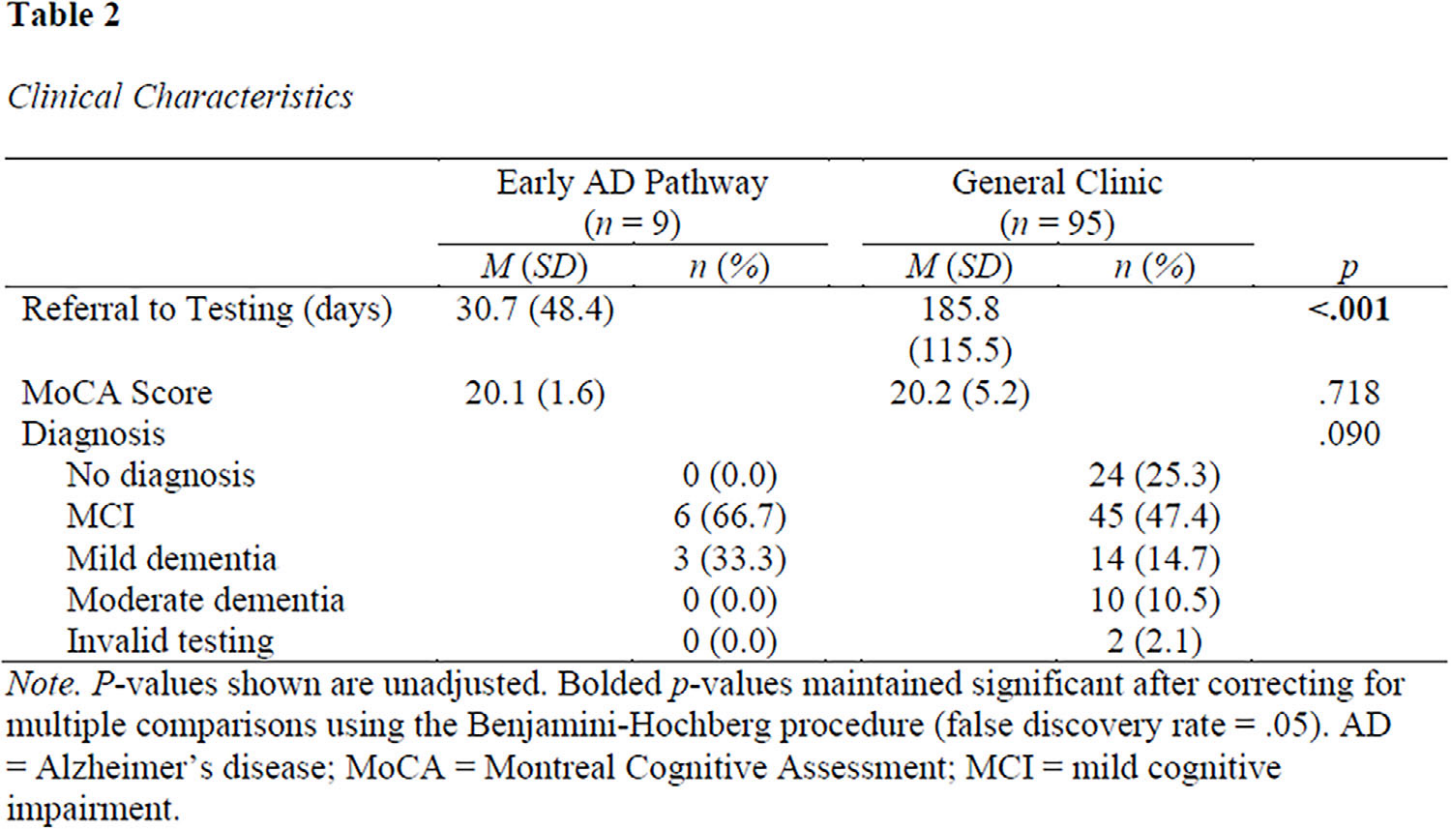# Meeting the demand: A pilot study to optimize service delivery for early Alzheimer’s disease using brief neuropsychological assessment

**DOI:** 10.1002/alz.086644

**Published:** 2025-01-09

**Authors:** Savana M. Naini, Ryan C Thompson, Kathleen Fuchs, M. Agustina Rossetti, Carol A Manning, Shannon Reilly

**Affiliations:** ^1^ University of Virginia, Charlottesville, VA USA

## Abstract

**Background:**

The increasing population of older adults and growing number of disease‐modifying therapies for Alzheimer’s disease (AD) highlight the need for timely differential diagnosis of neurodegenerative disorders despite high referral volumes. This study aimed to develop and pilot a brief neuropsychological battery to evaluate cognitive functioning in adults with suspected AD and improve service delivery by reducing the time between referral and diagnosis.

**Methods:**

Patients were referred to the “early AD pathway” by their neurologist or geriatrician after an initial evaluation in an outpatient multidisciplinary dementia clinic. Eligible patients scored 18‐25/30 on a brief cognitive screening measure (Montreal Cognitive Assessment [MoCA]), had an amnestic clinical presentation, and were thought to have mild cognitive impairment (MCI) or mild dementia. The “early AD pathway” involved chart review, a brief clinical interview, and an abbreviated clinical test battery based on the NACC Uniform Data Set (UDS) Version 3 neuropsychological battery. Patients were provided feedback shortly after the assessment, and referring providers followed up with patients to discuss additional neurodiagnostic workup and/or possible treatments. Nine patients referred for the “early AD pathway” pilot were compared to general memory/cognitive referrals seen by the neuropsychology clinic during fall 2023 (*n* = 95). Retrospective data collection included demographic characteristics and relevant clinical information, including the time between referral and evaluation, MoCA total score, and post‐evaluation diagnosis. Non‐parametric analyses were used to compare the “early AD pathway” patients to those undergoing traditional clinic procedures.

**Results:**

Preliminary results indicated significantly reduced wait times compared to traditional neuropsychological referrals (*M_diff_* = 155.17 days, *p*<.001) and successful identification of individuals with MCI or mild dementia. Moreover, 8/9 participants had an amnestic cognitive profile, suggesting high sensitivity for identifying a likely neurodegenerative process (i.e., suspected AD) in this pathway. Importantly, this pilot sample was small and racially homogenous, indicating the need for broader recruitment going forward.

**Conclusions:**

This pilot study demonstrated preliminary evidence for how to optimize neuropsychological service delivery to guide patients efficiently through an early AD identification process in a multidisciplinary clinic. This will be increasingly important as service demands and the number of pharmacological treatments increase over time.